# Wearable functional near infrared spectroscopy (fNIRS) and transcranial direct current stimulation (tDCS): expanding vistas for neurocognitive augmentation

**DOI:** 10.3389/fnsys.2015.00027

**Published:** 2015-03-09

**Authors:** Ryan McKendrick, Raja Parasuraman, Hasan Ayaz

**Affiliations:** ^1^Center of Excellence in Neuroergonomics, Technology, and Cognition (CENTEC), George Mason UniversityFairfax, VA, USA; ^2^School of Biomedical Engineering, Science and Health Systems, Drexel UniversityPhiladelphia, PA, USA

**Keywords:** HDtDCS, fNIRS, neuroergonomics, DLPFC, spatial working memory

## Abstract

Contemporary studies with transcranial direct current stimulation (tDCS) provide a growing base of evidence for enhancing cognition through the non-invasive delivery of weak electric currents to the brain. The main effect of tDCS is to modulate cortical excitability depending on the polarity of the applied current. However, the underlying mechanism of neuromodulation is not well understood. A new generation of functional near infrared spectroscopy (fNIRS) systems is described that are miniaturized, portable, and include wearable sensors. These developments provide an opportunity to couple fNIRS with tDCS, consistent with a neuroergonomics approach for joint neuroimaging and neurostimulation investigations of cognition in complex tasks and in naturalistic conditions. The effects of tDCS on complex task performance and the use of fNIRS for monitoring cognitive workload during task performance are described. Also explained is how fNIRS + tDCS can be used simultaneously for assessing spatial working memory. Mobile optical brain imaging is a promising neuroimaging tool that has the potential to complement tDCS for realistic applications in natural settings.

## Introduction

The rediscovery, over a decade ago (Nitsche and Paulus, 2000), of transcranial brain stimulation has led to a proliferation of research on brain and cognitive augmentation, both in healthy adults and in patients with neurological or psychiatric disease (Clark and Parasuraman, 2014). Augmentation refers to the improvement of cognitive functioning through task performance, or reversal of cognitive deficits that are normal consequences of performance in healthy adults (e.g., fatigue, stress) or those related to brain disorders. Ayaz et al., 2006; Hunter et al., 2013). Although the initial motivating rationale for the use of techniques such as transcranial Direct Current Stimulation (tDCS) was to develop alternative therapies for the treatment of neuropsychiatric diseases, augmentation effects were also seen in the healthy participants (Clark and Parasuraman, 2014; Flöel, 2014). These findings led to the current interest in developing methods of neurocognitive enhancement for healthy adults, for example to enhance human performance in complex tasks (such as air traffic control) or to accelerate skill acquisition in tasks (such as piloting unmanned vehicles) that typically require many hours or days of practice to master (Coffman et al., 2014; Parasuraman and Mckinley, 2014). Contemporary tDCS studies have provided a growing base of evidence for enhancing cognition through the non-invasive delivery of weak electric currents to the brain (Coffman et al., 2014). The main effect of tDCS is to modulate cortical excitability, depending on the polarity of the applied current. However, the underlying mechanism for the neuromodulation, such as how it is induced, how longs it persists, and the ways in which such modulation translate into improvement in performance are still not well understood and are currently the object of much research interest. Combining tDCS with multimodal neuroimaging techniques can enhance knowledge of its neuromodulatory effects in the brain (Hunter et al., 2013).

Traditional neuroimaging modalities such as functional magnetic resonance imaging (fMRI) have also been successfully utilized for studying cognition and understanding the neural mechanisms that contribute to the acquisition, development, and use of cognitive skills in artificial, controlled and stand-alone settings. These can be referred as read-only settings where functional neuroimaging is used to record brain activation and hence the flow of information is from brain to a computer. Moreover, modulation of neural signals can also be achieved through a neurofeedback training where a computer presents some derivative of the acquired brain signal in real-time back to user in visual or auditory form to establish the feedback loop (Hanslmayr et al., 2005; Gruzelier, 2009; Miller et al., 2010; Slagter et al., 2011; Ninaus et al., 2013). Neurofeedback training aims to allow volitional control of specific brain activity and has been extensively used in clinical neurorehabilitation or brain disorders such as attention-deficit hyperactivity, autism, epilepsy and mood disorders (Lubar et al., 1995; Hoffman et al., 1996; Raymond et al., 2005; Angelakis et al., 2007; Kouijzer et al., 2009; Lim et al., 2012; Heinrich et al., 2014). Neurofeedback training has been shown to enhance performance in cognitive tasks (Angelakis et al., 2007; Gruzelier, 2009) however, in this manuscript, we focused on tDCS based neuromodulation as it does not require training and has been utilized for human computer interaction applications (Clark and Parasuraman, 2014). Neuroimaging methods based on the MRI technique, such as functional MRI, resting state functional connectivity, and diffusion tensor analysis, have provided important information on the gray matter, white matter, and brain connectivity changes that accompany skill acquisition (Lewis et al., 2009; Lövdén et al., 2010; Voss et al., 2012; Strenziok et al., 2014), thus setting the stage for the development of theories of neuroplasticity, specifically for functional reorganization of neural networks and adaptation. For a review, see Elbert and Rockstroh (2004). However, some limitations of MRI are its requirement for participant immobility and its high operational cost. These factors have stimulated a need for lower-cost neuroimaging techniques that are portable and can be used in freely moving participants performing everyday tasks (Gramann et al., 2011, 2014). Among these are electroencephalography (EEG) and functional near infrared spectroscopy (fNIRS), both of which can be used for mobile brain imaging (Makeig et al., 2009; Gramann et al., 2011; Mehta and Parasuraman, 2013). The use of mobile brain imaging and stimulation techniques also falls within the field of neuroergonomics, defined as the study of the human brain in relation to performance at work and everyday settings (Parasuraman, 2003, 2011; Parasuraman and Rizzo, 2007). The main goal of neuroergonomics is to advance knowledge of brain functions in complex tasks and naturalistic work settings.

### Overview of Paper

The neuroergonomic approach has been considerably facilitated by the recent rise of development of portable and wearable neuroimaging devices, including EEG and fNIRS (Gramann et al., 2014). In this paper we review the potential uses of joint fNIRS and tDCS and describe wireless and battery operated fNIRS sensors (Ayaz et al., 2013) that provide new opportunities for brain and cognitive augmentation. We first briefly describe tDCS studies for enhancing skill acquisition in complex cognitive tasks. It is particularly important to assess and measure operator mental workload in situations where performance failures could result in catastrophic losses (e.g., military command and control, air traffic control, etc.). Improving operators’ cognitive abilities (such as working memory or attention) would help improve overall safety and productivity in such systems. Next we review tDCS studies that have targeted and assessed human operator performance. We then describe how fNIRS can be used to monitor brain dynamics during cognitive tasks, with a focus on evaluating effects on cognitive load. As a wearable and continuous monitoring sensor, fNIRS provides a safe and practical approach for monitoring brain activity in natural environments. We review studies that demonstrate task load related activity in the fNIRS signal. Next, we examine the combined use of fNIRS and tDCS for monitoring and enhancement of spatial working memory. Moreover, fNIRS + tDCS can realize new applications that were not possible before, such as “read-write” Brain Computer Interfaces (BCI) which can acquire (read) brain signals and also provide feedback directly to the brain (write) through stimulation. In general, optical brain imaging techniques such as fNIRS are a promising neuroimaging method and as the instruments continue to evolve, have the potential to become a complementary tool to tDCS for neuroergonomic applications in complex work tasks and in natural settings.

## Effects of tDCS on Complex Task Performance

Many noninvasive brain stimulation techniques for enhancing neurocognitive function exist, including transcranial magnetic stimulation (TMS) and tDCS (Clark and Parasuraman, 2014). In TMS an electric current is transiently passed through a magnetic coil positioned over the participant’s scalp over a brain region of interest. This creates a changing magnetic field that passes through the skull and induces current flow in the underlying cortical tissue sufficient to alter neural firing (Walsh and Pascual-Leone, 2005). tDCS involves application of a weak direct current (DC) electric current (1–2 mA) with electrodes attached to the scalp. A positive polarity (anode) is typically used to facilitate neuronal firing whereas a negative polarity (cathode) is used to inhibit neuronal firing. Application of tDCS is safe for experimental use in healthy participants for up to 30 min of stimulation (Bikson et al., 2009).

Understanding the mechanisms by which the tDCS modulations are induced and persist is still an open question. Initially, it was thought that application of weak DC current increases the resting neuronal membrane potential and thus lowers the threshold for firing of neurons (Bindman et al., 1964), but subsequent work suggests that other mechanisms are probably involved, such as dynamic modulation of synaptic efficacy (Rahman et al., 2013) and changes in neurotransmitter concentrations (Clark et al., 2011). Pharmacological tDCS studies also suggest neuronal membrane depolarisation during anodal stimulation may be responsible for the after-effects on cortical excitability (Liebetanz et al., 2002).

tDCS can be applied to better understand brain mechanisms and their relation to cognitive processes, although tDCS is not as focal in activating or inhibiting brain regions in comparison to TMS given the diffusivity of current flow for anode over region of interest, extra-cephalic cathode montages (“ring” montages; anode over region of interest encircled by multiple cathodes provide more focal stimulation but not to the level implemented by TMS (Datta et al., 2009)). Recent tDCS studies have allowed researchers to make inferences regarding the neural basis of learning, memory, perception, and motor actions (Filmer et al., 2014). The study by Holland et al. (2011) investigated language function of healthy participants and aimed to help develop the approach for potential clinical deployment for rehabilitation of brain-damaged patients. Authors utilized fMRI to monitor and localize the effects of tDCS stimulation concurrently. Left frontal anodal tDCS was used during an overt picture-naming task and results provided important evidence of contribution of the left inferior frontal cortex in the naming task and identified Broca’s area for tDCS based rehabilitation (Holland et al., 2011).

Another example is a study by Clarke et al. (2014) in which the role of dorsolateral prefrontal cortex in attention bias modification (ABM) was investigated. The exaggerated attention to mildly threatening conditions is defined as the attention bias to threat and has been reliably observed across a range of anxiety and mood disorders. Reducing attention to threat in high anxiety patients has been demonstrated to also reduce anxiety symptoms, and thus suggests the promise of treatment of anxiety pathology. The authors utilized tDCS to isolate and test fMRI findings reported earlier by Browning et al. (2010) which implicated lateral prefrontal cortex in inhibitory control of attention in relation to threatening information. This study by Clarke et al. (2014) demonstrated the complementary nature of neuroimaging and neurostimulation (as the finding verified the functional MRI results of Browning et al. (2010)); and, highlights the potential power of joint neuroimaging and neurostimulation for novel interventions while establishing a broad neurocognitive framework. Below we further examine such joint investigations by combining tDCS and fNIRS.

Many studies have found that stimulation of different brain regions with tDCS can enhance performance of basic cognitive tasks that recruit the corresponding brain regions. For example, stimulating the dorso-lateral prefrontal cortex (DLPFC), which has been shown in neuroimaging studies to be involved in working memory, accordingly enhances performance on working memory tasks (Fregni et al., 2005). Beyond working memory, tDCS has also been found to enhance learning and performance on a wide variety of perceptual, cognitive, and motor tasks (for reviews, see Jacobson et al. (2012) and Coffman et al. (2014)). Here we provide a few examples of the effects of tDCS on more complex tasks representative of work settings.

One example involves surveillance and security operations, as in threat detection (Parasuraman and Galster, 2013). Accurate and timely detection of obscured or concealed objects, or the actions and movements of other people, is a critical need in many such work environments, both in the military and in civilian organizations. Skill in such threat detection tasks typically develops only after extensive training lasting many days. Can the development of expertise be speeded up with tDCS? Recent studies provide a positive answer (Clark et al., 2012; Falcone et al., 2012). These studies involved use of a complex task requiring participants to watch videos of naturalistic scenes containing movements of soldiers and civilians. Still images were extracted from the videos and manipulated so that half were targets, defined as concealed objects (e.g., bombs), people engaging in threatening activity (e.g., snipers), and so on, whereas the same scene without the threat was a non-target. An fMRI study was first conducted to determine optimal sites for application of tDCS (Clark et al., 2012). A total of 104 participants volunteered for the study and were imaged as novices. A subset, 13 participants performed the task during fMRI data collection to identify the brain networks supporting the identification of concealed objects and changes with learning. The results indicated that the right inferior frontal gyrus was the major locus of a distributed brain network that mediated acquisition of the threat detection task and so was chosen as the optimal stimulation site.

Falcone et al. (2012) examined whether tDCS applied to this location enhanced perceptual sensitivity in threat detection. Participants were given four training blocks of and were required to indicate whether a threat was present or absent. Two test blocks were given before training and were similar to training blocks, except that no feedback was given after each response. Anodal tDCS was applied to the electrode site F10 in the EEG system, over the right sphenoid bone, corresponding to an area overlying the inferior frontal gyrus. Although this is not as precise as subject-fMRI guided location selection, anatomical landmarks using international 10–20 system provided a viable solution which was confirmed by the results of the study. The cathode was placed on the contralateral (left) upper arm. Participants were randomly assigned to either active (2 mA current) or sham stimulation (0.1 mA) for a total of 30 min during the first two training blocks.

Compared to the 0.1 mA sham stimulation control, 2 mA stimulation increased perceptual sensitivity in detecting targets and accelerated learning. Performance was near chance (*d*′ = 0) in both groups at the beginning of training. However, skill acquisition with tDCS was both rapid and extensive: On completion of training, participants in the active stimulation group had more than double the *d*′ of the control group. There were no group or training effects on the response bias measure β, indicating that tDCS improved the actual efficiency of threat detection. Furthermore, threat detection sensitivity remained at a high level immediately after training and, more importantly, 24 h later. This last finding bodes well for the use of tDCS as a training method with potentially lasting effects in naturalistic work tasks.

A second example involves intelligence analysis, McKinley et al. (2013) trained image analysts to find and correctly identify ground targets, such as tanks and surface-to-air missile launchers, in synthetic aperture radar imagery. Stimulation of the right frontal cortex, using the same anodal F10 scalp location (cathode on the contralateral bicep) as in the previously described study of Falcone et al. (2012), significantly improved object recognition learning rates. During the first phase of training, one group was given active tDCS for 30 min; another, sham tDCS (active tDCS for 30 s); and a third group, no tDCS. Participants were then given a second round of training with the stimulation conditions reversed (i.e., the active tDCS group switched to sham tDCS, whereas the sham tDCS group received active tDCS in the second round). Both groups experienced larger increases in target acquisition accuracy when given active tDCS when compared to sham or no stimulation in either session. The image analysis task also included a change detection task in both training sessions. After the target image was complete, one of the targets (randomly assigned) changed in orientation, position, target type, or disappeared completely. Change detection performance was improved only when tDCS was applied in the second session. Thus, tDCS aided in change detection only after the analyst gained some experience with the images and target types. A similar finding was reported by Coffman et al. (2012), who found that tDCS had a larger effect on threat detection for images that had been viewed previously. These findings may reflect tDCS-induced plasticity changes in the brain networks responsible for object encoding and retrieval.

These are just two examples of the effectiveness of tDCS as a neuroergonomic tool for accelerating skill acquisition in complex, work-relevant tasks. Other examples are reviewed by Parasuraman and Mckinley (2014). Prior neuroimaging evidence suggests that such performance gains probably resulted from activation of specific brain networks associated with the relevant cognitive functions. However, direct evidence of modulation of brain dynamics would provide stronger evidence for such an association. Below, we examine how the combined use of fNIRS and tDCS can help in this endeavor. We begin, however, with a brief overview of the use of fNIRS alone in studies of cognitive workload.

## Using fNIRS to Monitor the Relationship of Cognitive Workload and Brain Dynamics

fNIRS provides an attractive method for continuous monitoring of brain dynamics in both seated or mobile participants. fNIRS is safe, highly portable, user-friendly and relatively inexpensive, with rapid application times and near-zero run-time costs (Villringer and Chance, 1997; Ferrari and Quaresima, 2012). The most commonly used form of fNIRS uses infrared light, introduced at the scalp, to measure changes in blood oxygenation as oxy-hemoglobin converts to deoxy-hemoglobin during neural activity, i.e., the cerebral hemodynamic response. fNIRS uses specific wavelengths of light to provide measures of cerebral oxygenated and deoxygenated hemoglobin that are correlated with the fMRI BOLD signal (Cui et al., 2011; Sato et al., 2013). Below we briefly review fNIRS studies of cognitive workload.

For objective measures of cognitive workload in naturalistic environments, fNIRS offers a number of advantages over other measurement techniques such as fMRI. In particular, the high operational costs of fMRI makes long-duration or longitudinal (e.g., training) studies impractical. Cost is less of an issue with fNIRS as the systems themselves are less expensive and once purchased require no extra costs to run. fNIRS also does not require the participant to be immobile and the use of wireless fNIRS allows for imaging brain dynamics during tasks that require a participant to move regularly, as in motor and other physical tasks (Mehta and Parasuraman, 2014) and in naturalistic settings (Ayaz et al., 2013). fNIRS also offers a compromise between the spatial resolution of fMRI and temporal resolution of EEG. The superior spatial resolution (localization of activation) of fNIRS relative to EEG allows for greater accuracy in identifying specific brain regions responding to changes in workload. The superior temporal resolution (higher sampling rate) of fNIRS relative to fMRI affords improved statistical power when analyzing changes in the shape of the hemodynamic response.

fNIRS has proven beneficial for measuring workload in a number of complex tasks. Examples include supervisory control, natural orifice surgery simulations, and driving. In a study of air traffic controllers, Ayaz et al. (2012) found that as the number of supervised aircraft increased there was an increase in cerebral oxygenation (oxygenated hemoglobin minus deoxygenated hemoglobin) in the left medial/orbito frontal cortex. The relationship was linear and corresponded with increased oxygenation observed in the same sample during a multi-load N-back working memory task (Ayaz et al., 2012). Similarly during natural orifice translumenal endoscopic surgery (NOTES) simulation experienced surgeons familiar with NOTES showed increases in oxygenated hemoglobin in bilateral ventral lateral prefrontal cortex (VLPFC) when the simulation required a more difficult navigation path through an orifice (James et al., 2011).

fNIRS measurement of mental workload has also been used within the context of driving. In two separate studies while individuals drove on a closed road it was observed that deceleration increased oxygenated hemoglobin in regions involved in eye movements and optic flow (Yoshino et al., 2013a,b). The results indicated that deceleration is more cognitively taxing on visual processing than acceleration or constant velocity driving. Increases in oxygenated hemoglobin in bilateral VLPFC during U-turns was also observed (Yoshino et al., 2013b), suggesting the need for increased executive control relative to acceleration, deceleration, and constant velocity driving. Other recent studies also demonstrated the potential of fNIRS for assessment of cognitive workload (Abibullaev and An, 2012; Naseer and Keum-Shik, 2013; Afergan et al., 2014; Bogler et al., 2014; Derosière et al., 2014; Herff et al., 2014; Schudlo and Chau, 2014; Solovey et al., 2015).

Although a linear relationship between task workload and hemodynamics has often been observed (Ayaz et al., 2012; Fishburn et al., 2014) where the difficulty of the task at hand does not exceed the cognitive capacity of participant, whereas when cognitive capacity is exceeded the observed effects on hemodynamics conform to the shape reported by the Yerkes–Dodson law (Yerkes and Dodson, 1908). On a supervisory control task a negative quadratic relationship (inverted U) between workload and DLPFC activation was found (Durantin et al., 2014). Individuals were asked to control remotely operated vehicles as they navigated through an airspace while avoiding no fly zones. Workload was manipulated by altering crosswinds, vehicle inertia and memory load regarding supervisory control. It was also noted that there was a strong correlation between increased DLPFC activation in the highest workload condition and performance. This actually suggests that workload alone does not have a quadratic relationship with functional hemodynamics, but instead once mental overload is reached functional activation decreases. Evidence from two other studies supports this claim. Yamauchi et al. (2013) had participants play a modified version of “rock, paper, scissors” against a computer, with the objective to actually lose each hand. The computer presented one of the three hands and the participant had to choose the losing hand. Workload was manipulated by decreasing the inter stimulus interval (ISI). Furthermore these decreases were adapted to each participants minimum effective ISI. When workload was manipulated as a function of an individual’s maximum workload, only linear increases in oxygenated hemoglobin were observed in left lateral prefrontal cortex, premotor cortex and supplementary motor area (Yamauchi et al., 2013).

Similarly in a dual-working memory training study when task memory load increased as a function of participant’s skill acquisition, a strong linear increase in total hemoglobin after an initial decrease in activation occurred while participants adapted to the task. However a different group of participants had their task memory load yoked to the performance of the other group, and they showed a negative quadratic relationship between memory load and total hemoglobin (McKendrick et al., 2014). Taken together these findings suggest that the presence of a negative quadratic slope during fNIRS monitoring of workload dynamics is indicative of task overload. This trend can be used to assess the points at which overload occurs for individuals, or as a means of ensuring that tests of workload only include load up to an individual’s maximum effective capability. This can be used to optimize operator work periods, introducing adaptive automation (Byrne and Parasuraman, 1996), or delegation of tasks to other operators as methods of optimizing operator efficiency and system performance. It is also apparent that an individual’s maximum effective workload can change with task training. Therefore this trend may occur concurrently with an individual’s skill acquisition and non-linear components should be included within statistical models of workload dynamics to observe and utilize this quadratic trend.

The changes in oxygenated and deoxygenated hemoglobin representative of mental workload may not only arise from cognitive work. Both physical and emotional work can affect and potentially invalidate measures of cognitive workload. Submaximal physical effort can reduce mental performance, furthermore increasing submaximal physical effort has similar effects on oxygenated and deoxygenated hemoglobin as moving from a single cognitive task to a dual cognitive task (Mandrick et al., 2013). Mental and physical work to exhaustion may also cause cognitive interference resulting in decreased oxygenated and increased deoxygenated hemoglobin in prefrontal cortex (Mehta and Parasuraman, 2013).

## Monitoring the Effects of tDCS on Brain Dynamics Using fNIRS

There is now considerable evidence that tDCS can boost brain plasticity processes and accelerate skill acquisition in complex cognitive tasks (Clark and Parasuraman, 2014). Less well known, however, is the neural changes that make such performance gains possible. There are only a few investigations of simultaneous neuroimaging and stimulation studies, such as using fMRI (Alon et al., 2011; Antal et al., 2011; Holland et al., 2011; Kwon and Jang, 2011). However, the electric current flow of tDCS can create confounds in simultaneous fMRI echo-planar imaging (Antal et al., 2014). For a review of neuroimaging artifacts and limitations during simultaneous tDCS and fMRI, see Saiote et al. (2013) and Antal et al. (2014). Hence, a neuroimaging tool is needed that is inherently independent of electrical stimulation. As an optical imaging technique fNIRS provides one such neuroimaging approach.

Combining fNIRS with tDCS can provide some insights for understanding brain plasticity associated with skill acquisition. An initial basic research direction for joint use of fNIRS and tDCS is for understating how tDCS effects the brain in both animal models (Han et al., 2014) and human studies (Merzagora et al., 2010; Khan et al., 2013; Muthalib et al., 2013; Ishikuro et al., 2014; Jones et al., 2015). Merzagora et al. (2010) reported on the anterior prefrontal cortex effects of tDCS before and after stimulation using a prefrontal sensor pad based fNIRS measurement. Results indicated that fNIRS successfully captured the activation changes induced by the tDCS stimulation. Khan et al. (2013) compared altered hemodynamic patterns in the sensorimotor cortex in response to bi-hemispheric tDCS polarities and their relationship to muscle activity and motor task performance. Muthalib et al. (2013) utilized anodal tDCS based motor cortex stimulation to study neuromuscular fatigue and task failure related prefrontal cortex activation measured by fNIRS. Ishikuro et al. (2014) studied the relationship between frontal and sensorimotor cortices and motor learning of tasks used in rehabilitation. Healthy participants performed the task using a whole head fNIRS system. The neuroimaging session was used to identify relevant brain area (anterior dorsomedial prefrontal cortex) for stimulation in a separate experiment. Participants performed the same task with and without tDCS. Authors reported significant effects of tDCS and improvement in performance with stimulation. Jones et al. (2015) investigated the role of motivation (incentives) and tDCS in improving performance for both high and low working memory capacity participants. Authors used fNIRS to assess the cortical effects and brain activity changes.due to tDCS stimulation. The underlying motivation of such joint stimulation and neuroimaging studies is to extend boundaries of knowledge on brain-behavior relationships and translate the acquired knowledge for potential clinical and neuroergonomic applications.

### Combined tDCS-fNIRS: Neuroergonomics Pilot Study

We illustrate the utility of the combination of simultaneous tDCS and fNIRS techniques in a study examining the effects of tDCS on spatial working memory. The task involved recalling the location of 5–7 randomly spaced black disks on a computer display after a short retention period. Each trial began with 15 s of fixation followed by a 1 s presentation of 5–7 randomly spaced black disks. A 4 s random noise mask was displayed after the presentation of the stimulus, after which participants were instructed to respond and recall the number and positions of the stimulus. A more complete description of the task is presented in McKendrick et al. (2014).

Participants received a block of baseline trials, followed by two blocks of sham stimulation, one block of 1 mA stimulation using a high-density tDCS montage, and a final block of continued stimulation monitoring, with each block consisting of 33 trials. Participants were fitted with an elastomere cap with high density tDCS (HD-tDCS) electrode holders positioned at F2 and F10 in the 10–20 EEG system. A Ag/AgCl sintered ring electrode was placed in each holder along with electroconductive gel to conduct the current to the scalp (See Villamar et al., 2013 for a more detailed description of the Soterix HD-tDCS system). An fNIR Devices Model 1100 NIRS imaging device sensor that has 16 optodes (10 photodetectors and 4 light emitters each using 730 nm and 850 nm wavelengths of light) was attached to the forehead for monitoring changes in frontal oxygenated and deoxygenated hemoglobin (for a more complete description of the fNIR Devices Model 1100 NIRS see Ayaz et al., 2012).

**Figure 1:**
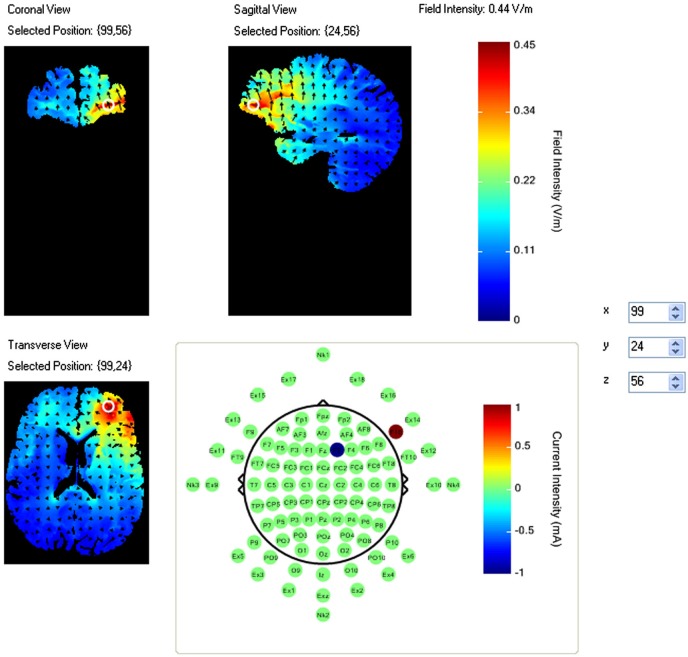
**Current flow model of tDCS montage (F10 anode, F2 cathode), field intensity of 0.44 V/m represented at white ring in coronal, sagittal and transverse views**. Arrows represent direction of current flow.

A pilot study using this task had identified a region of right VLPFC that showed an increase in activity during the task period relative to fixation (McKendrick et al., 2014). This region also showed a correlation between increased performance and increased neural efficiency (increased performance negatively modulated increases in activity amplitude). As such this region was selected for stimulation via HDtDCS and is an example of fNIRS guided tDCS. Using the modeling software HD-explore (Soterix Medical), we constructed a montage that elicited maximum current flow to right VLPFC (Figure 1) by placing the anode at F10 and cathode at F2.

The current density of this montage is considerably higher than that traditionally observed with two electrode montages using saline soaked sponges. For this reason twice during the study participants were asked to report the current severity of sensations such as heat, tingling, and itching. No participants were removed from the study due to reports of severe sensations.

Linear mixed effects models were used to assess changes in task performance as a function of time and stimulus condition. Analysis was performed in R with package “LME4” and function “lmer”. Model selection and control for over fitting were done with AIC and BIC log-likelihood weighting functions with BIC taking precedence if the two weighting functions selected a different model.

Raw NIRS time series data were low pass filtered and corrected for motion artifacts, after which relative concentrations of oxygenated and deoxygenated hemoglobin were calculated with the modified Beer Lambert law, with the first 10 s of fixation for a given block of trials used as the NIRS baseline. Oxygenated and deoxygenated hemoglobin time series for each optode were analyzed with linear mixed effects regression. Orthogonal regressors were constructed with boxcar time series representing hypotheses of interest and convolved with a canonical hemodynamic response function. The following regressors were constructed and entered into a design matrix: (1) increased activity during the task period relative to fixation; (2) correlation between task activity amplitude and performance; (3) increased task amplitude during stimulation relative to baseline; (4) increased task amplitude during stimulation relative to sham; (5) correlation between task performance and increased task activity during stimulation relative to baseline; and (6) correlation between task performance and increased task activity during stimulation relative to sham. Fixed effects were composed of the full design matrix of regressors and random effects were selected via the same methods used for behavioral model selection. Multiple comparisons were corrected for using the Hochberg false discovery rate correction. Final effects of increases or decreases in activation were determined by comparing the sign of beta coefficients for significant changes in oxygenated and deoxygenated hemoglobin. Opposite signs of oxygenated and deoxygenated hemoglobin, where the beta coefficient of oxygenated hemoglobin was positive were interpreted as increases in activity; where the coefficient of oxygenated hemoglobin was negative were interpreted as decreases in activity.

The most parsimonious behavioral model specified fixed effects of a linear and quadratic effect of experimental block, and random effects of participant intercept uncorrelated with experimental block. There was a significant linear effect of block (*b* = −0.38, SE = 0.16, *p* < 0.05). This suggests that increased time on the task lead to a decrement in performance. However there was also a significant quadratic effect of block (*b* = 0.06, SE = 0.02, *p* < 0.05). This positive quadratic effect counteracted the decline in performance following the first three blocks. The uptrend in performance also corresponds with the time at which participants began receiving stimulation, and continues even after stimulation was removed.

**Figure 2:**
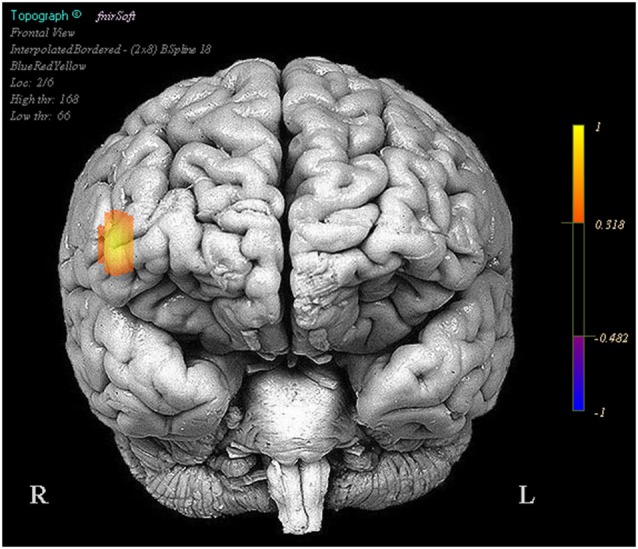
**Regions in which effects represent a correlation between increased activity and increased task performance**. Legend represents the presence and direction of the effect, not *p* or *t* values.

In the NIRS data there were a number of optodes that showed significant effects in oxygenated and deoxygenated hemoglobin for the design matrix regressors we constructed; however for the sake of brevity and clarity only effects where oxygenated hemoglobin and deoxygenated hemoglobin had opposite beta coefficients are reported. Visualization of brain activation patterns are described elsewhere (Ayaz et al., 2006) and more information on placement of optodes, see Ayaz et al. (2012). Final models for each optode consisted of the full design matrix for fixed effects and random effects were participant intercept uncorrelated with time. There were no significant activation changes comparing task period to fixation, however optode 16 in coherence with our pilot findings showed a relationship between activity amplitude during the task period and subsequent performance (Oxy *b* = 0.019, SE = 0.008, *p* < 0.05, Deoxy *b* = −0.008, SE = 0.004, *p* < 0.05) (Figure 2).

**Figure 3:**
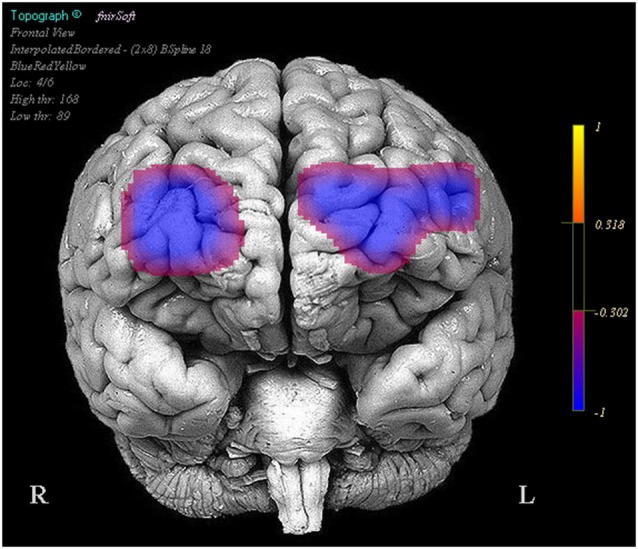
**Regions in which effects represent a decrease in activity during stimulation trials relative to the sham trials**. Legend represents the presence and direction of the effect, not *p* or *t* values.

Optodes 1, 3, 5, 6, 7, 11, 12, and 13 showed evidence of increased activation during the task period for stimulation blocks relative to the base line block. However these same regions and optode 14 showed a decrease in activation during the task period when comparing the stimulation blocks to the sham blocks (Figure 3).

**Figure 4:**
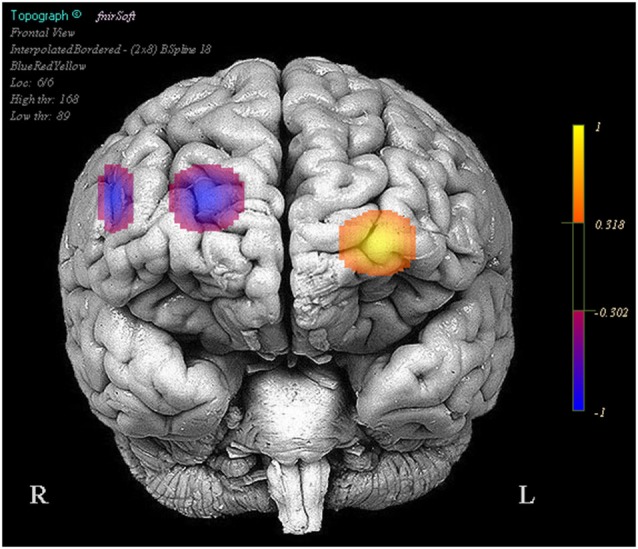
**Regions in which effects represent a correlation between activity and increases in performance in stimulation trials relative to the sham trials**. Legend represents the presence and direction of the effect, not *p* or *t* values.

We also observed that reduced positive activity in optodes 1 and 13 were associated with higher performance between the stimulation and baseline trials. Finally greater reduced activity in optodes 11 (Oxy *b* = −0.058, SE = 0.011, *p* < 0.001, Deoxy *b* = 0.022, SE = 0.005, *p* < 0.001) and 15 (Oxy *b* = −0.118, SE = 0.014, *p* < 0.001, Deoxy *b* = 0.043, SE = 0.009, *p* < 0.001) was associated with improved performance between the stimulation and sham trials, this was also accompanied by less negative activity in optode 6 (Oxy *b* = 0.027, SE = 0.012, *p* < 0.05, Deoxy *b* = −0.017, SE = 0.007, *p* < 0.05) correlating with improved performance (Figure 4).

1 mA of DC was applied with anode at F10 and cathode at F2 while participants performed a spatial memory task while being concurrently monitored with fNIRS. Task performance declined rapidly following baseline, possibly reflecting changes in vigilance or fatigue. However this decrement was overcome and almost eliminated following HDtDCS stimulation. The stimulation also had a number of effects on hemodynamic correlates of neural activity and their relationship to task performance. Specifically stimulation reduced the activity in bilateral prefrontal cortex, however most of these changes were unrelated to the effect of tDCS on task performance. Only continued decreased activity in right dorsal medial (optode 11), and right dorsolateral PFC (optode 15) were associated with the increase in performance experienced as participants shifted from the sham blocks to the stimulation blocks. This is particularly interesting as the cathode was placed directly above right dorsomedial PFC at the sight associated with performance recovery. Furthermore it is interesting to note that the region selected for modulation via our model of current flow was not actually modulated by stimulation, however its activity was still consistently associated with task performance. Taken together these results suggest that tDCS can modulate the neural activity of specific brain regions near the site of stimulation, however current models and protocol for determining tDCS montages are lacking, as it appears there are intimate interactions between stimulation montage, task and underlying hemodynamics that are complex. Additional joint tDCS and fNIRS studies are needed to further unravel these complexities and to better define the pattern of cortical excitation induced by tDCS during the performance of cognitive tasks.

## Wireless Brain Imaging With tDCS

Significant progress has been made over the last decades in understanding the brain physiology and neural dynamics related to cognitive processes and behavior. However traditional neuroimaging tools such as fMRI severely restrict subject movements due to the inherent imaging operation (Makeig et al., 2009). Such technical limitations require brain imaging in more artificial settings separated from dynamic and multi-faceted natural environment (Gramann et al., 2014). To be able to capture brain dynamics related to natural cognition, mobile brain imaging systems are needed to operate in complex and partially unpredictable environments, consistent with mobile brain/body imaging (MoBI) and neuroergonomics approaches (Gramann et al., 2011; Parasuraman, 2011), A new generation of portable brain sensing technologies of EEG and fNIRS have begun to overcome the limitations of traditional neuroimaging through untethered measurements and wearable sensors (Liao et al., 2012; Ayaz et al., 2013; De Vos et al., 2014; Stopczynski et al., 2014; Mihajlovic et al., 2015). For a review of commercial available mobile EEG systems see (Mihajlovic et al., 2015) and recent studies demonstrated combined EEG and tDCS (Faria et al., 2012; Schestatsky et al., 2013; Mangia et al., 2014) as well as the combined fNIRS and tDCS studies (Khan et al., 2013; Ishikuro et al., 2014; Jones et al., 2015). However, joint use of EEG and tDCS is prone to artifacts, requires additional effort (such as extra reference electrodes and processing) to control and isolate the electrical fields to prevent contamination. Since fNIRS is optical (no electrical interference) and fNIRS sensor usually has an opening directly over the measurement area (light source and detectors are positioned around the measurement area, see Figure 6) there’s a natural opportunity for integration. Potential applications of portable fNIRS were reviewed recently for neuroergonomics (Ayaz et al., 2013) and economics research (Kopton and Kenning, 2014). These developments have provided an opportunity for coupling mobile brain imaging sensors with wireless tDCS for monitoring and modulating brain activity in ecologically valid natural environments.

**Figure 5:**
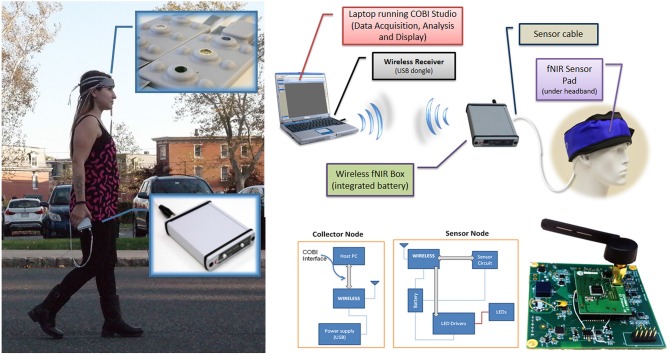
**Wireless fNIRS System**. (left) Battery operated and wireless unit allows untethered outdoor measurement (right, up). Block diagram of the overall system (right, bottom), Building blocks and circuit representation (Ayaz et al., 2013).

**Figure 6:**
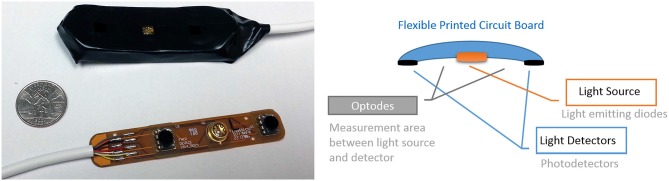
**Miniaturized and scalable fNIRS sensor pad with 2 optodes can be integrated with electrodes**. (left) prototype sensor pad circuit board and covered with foam enclosure. A U.S. quarter is included for size. (right) The 2 optodes sensor pad parts.

Recent comprehensive reviews on fNIRS technology (Ferrari and Quaresima, 2012) confirm that the vast majority of instrumentation development has been conducted on continuous wave (CW) type fNIRS. CW systems have a limitation in terms of their information content (i.e., it measures only changes of oxy and deoxy-Hb) compared to frequency and time-resolved fNIRS systems. However, CW fNIRS is also most appropriate for miniaturization and portable system development, because the signal type and acquisition timing requirements are less demanding.

The development of wearable and low cost fNIRS systems began in 1990s and by Chance et al. (1997) specifically for prefrontal cortex brain hemodynamics and muscle measurements. These systems, were later further developed into the portable systems at Drexel University for functional brain imaging using both desktop and miniaturized wireless versions (Ayaz et al., 2013) as well as breast tumor scanning (Sao et al., 2003), chronic wound monitoring (Weingarten et al., 2008) and brain hematoma scanning (Ayaz et al., 2011a).

fNIRS based wireless brain imaging systems have also been of interest and Hoshi (2003) reported use of one the earliest for assessing regional blood flow related to emotion in children. The system had one detector and two light sources, providing two optodes overall. Participants were carrying the equipment and transmitter in a backpack. Later, Yurtsever et al. (2006) reported a pocket PC integrated system that reduced the overall size considerably by using off-the-shelf embedded system as computational platform and featured up to 48 channels (16 optodes). Also, Muehlemann et al. (2008) described an *in vivo* measurement system that featured wireless data transfer with up to 32 channels and high sampling rate to reach fast optical signal. Holper et al. (2010) used that system for virtual reality based neurorehabilitation approach during observation and motor imagery tasks. Also, an EEG integrated prototype has been used in epilepsy research (Safaie et al., 2013). More recently, Muthalib et al. (2014) presented an HD-tDCS EEG/fNIRS capable experiment setup that was used for studying both electrophysiological and hemodynamic components of the modulation of cortical sensorimotor networks.

In previous work we have reported a custom miniaturized system (Rodriguez and Pourrezaei, 2011) that can be used for general purpose functional neuroimaging studies of prefrontal cortex (Ayaz et al., 2013). The device is a smart-phone size unit that can be carried in hand (See Figure 5), and drive up to 5 optodes (15 channels) at 4 Hz sampling rate. The system interfaces and transmits data wirelessly to a PC that runs the COBI Studio (Ayaz et al., 2011b). The implemented system is depicted in Figure 5 below. The main advantage is further miniaturization of the hardware unit and hence no need for backpack, subjects can carry the system in their pocket or hand allowing more freedom in experimental design.

Integration of fNIRS sensors with tDCS also shows promise given that optical brain imaging is not influenced by electrical stimulation. Simultaneous use with fiber based fNIRS sensors is less of an issue as placement of fibers that run perpendicular to scalp leaves much space for other types of sensors. However, such sensors require laser light sources and larger hardware equipment, which are not as portable as LED based systems. Hence further miniaturization and customization of LED based sensor pads is needed. Figure 6 below depicts a miniaturized prototype fNIRS sensor pad that is compatible with the fNIRS wireless unit described above (Ayaz et al., 2013) and a similar configuration was already tested with tDCS (Rodriguez and Pourrezaei, 2011). The combined tDCS fNIRS in that study was constructed by first molding the insulated fNIRS PCB in a skin safe silicon cast which was designed to hold tDCS electrodes in standard size (2″ × 3.5″) acting as a sleeve to the electrode. Systemic performance tests with varying power and gain parameters indicated that undesired interference is not introduced by the tDCS stimulation and that the fNIR sensor performs as expected. Similarly, there are also prototype fNIRS sensors that are already integrated with EEG electrodes for hybrid measurements (Lareau et al., 2011; Leamy et al., 2011; Safaie et al., 2013).

Since the fNIRS sensor positioning of the light source and detectors are around the measurement area (which is in between the light source and detector as illustrated in Figure 6) and not directly on top of the measurement area, combining with tDCS is feasible and practical from a hardware development perspective. Moreover recent developments in tDCS systems provide multi-channel tDCS systems that allow independent control of individual electrode currents, such as the HDtDCS systems developed by Soterix Medical and the wireless tDCS system Starstim (by NE Electrics) enabling potential ambulatory experimental protocols.

## Future Directions

This paper reviews the potential joint use and future convergence of two technologies for neuroimaging and neurostimulation, fNIRS and tDCS, and how the two can be synergistically used together to enhance our current understanding of brain dynamics. Both technologies have complimentary capabilities, and both are built wearable and wireless that allow for application in natural environments and real world settings. Future neuroergonomics applications could range from enhanced/accelerated learning and training of complex human-machine systems to optimization of task load for improved safety and productivity.

Also, joint use of tDCS and fNIRS could enable new unique applications such as read-write BCI. A BCI is defined as a system that captures and transforms signals originating from the human brain into commands that can control external applications or instruments. In its most general form BCI provides a route for neural output that does not involve the neuromuscular system (Wolpaw et al., 2002; Lebedev, 2014). BCI systems have a wide range of potential applications, including rehabilitation and assistive use for severely paralyzed patients to help them communicate and interact with their environments, as well as monitoring brain activity for assessment of mental state or intervention in various psychiatric conditions and/or to augment the interactivity of healthy individuals.

Current noninvasive BCI systems are read-only as they capture brain activity and produce output/action for user. However, future portable and noninvasive BCI systems can also write to brain for direct communication and bypassing the peripheral nervous system and enhancing the brains’ sensory input mechanism. Earlier studies in animal models achieved meaningful sensorimotor information in real time using invasive intracortical microstimulation to deliver sensory feedback signals in rats (Pais-Vieira et al., 2013) and monkeys (O’Doherty et al., 2011). This concept has been tested on humans recently after lab prototypes and demonstrations indicated feasibility and Grau et al. (2014) published their approach for Brain to Brain Communication which was made possible with dual use of noninvasive neuroimaging and neurostimulation. In the study, authors utilized EEG for capturing voluntary motor imagery related activations which were relayed as light perception to second brain by stimulating occipital lobe via TMS. Practical brain to brain communication would have profound impact on how we communicate and work, and as a portable system, tDCS is the natural candidate for closing the loop for future portable BCI systems.

As the potential use of future BCI systems has implications from individual to society at large, ethical aspects have also been a focus of discussion as part of the rising field of neuroethics (Illes and Bird, 2006; Haselager et al., 2009; Schermer, 2009; Clausen, 2011; Nijboer et al., 2011; Vlek et al., 2012). One of the immediate concerns is related to “treatment vs. research” which is related to the decision of using new systems on clinical and vulnerable populations such as locked-in patients. As in all new medical technologies, clinical utility and benefit vs. the risk (e.g., when using invasive neuroimaging or burden of engaging with the system) has to be evaluated with due process (informed consent) (Vlek et al., 2012). Also, privacy has been a core concern (Nijboer et al., 2011; Fairclough, 2014) and mostly attributed to keeping ones’ physiological signals private. With the influence of contemporary science-fiction, write-only or read-write BCI have often been considered akin to mind control. Writing to the brain has been used here in terms of modification/modulation of brain signals and is a physiological effect with immediate clinical uses (e.g., Parkinson treatment with deep brain stimulation). Current concepts of write-only or read-write BCI can only operate with the user’s consent and engagement. And, the design of future BCI systems should be informed by neuroethics considerations from personal to societal perspectives. For a discussion of the near and long-term issues please see recent reviews by Clausen (2011), Nijboer et al. (2011), Vlek et al. (2012) and Attiah and Farah (2014).

Another interesting future direction could be the unification of neuroimaging and neurostimulation technology by using near infrared light. A novel integration of optics and genetics is the emerging field of optogenetics which uses light to control neurons that have been genetically modified to be sensitive to light (Deisseroth, 2011). Optogenetics studies has been exponentially growing to observe and perturb neural mechanisms from single cell level to animal brain models. The requirement of genetically encoded, protein-based probes to achieve experimental manipulation is a major limitation for human studies. Optical stimulation with near infrared lasers that are low powered but have high energy density could be a solution (Wells et al., 2005a,b; Shapiro et al., 2012). A recent study suggest that such lasers could be utilized to excite cells by changing their electrical capacitance (Shapiro et al., 2012). Although light sources for such lasers would be different for reading and writing, having a unified/fused wearable pad that can both record and stimulate brain activity could enable new applications in natural environments. In summary, the simultaneous use of tDCS and fNIRS, the development of wireless, portable fNIRS systems, and the potential development of optical systems for both stimulation and neuroimaging are opening up new vistas for neurocognitive augmentation, with exciting new clinical and neuroergonomic applications.

## Disclosure

fNIR Devices, LLC manufactures the optical brain imaging instrument and licensed IP and know-how from Drexel University. H. Ayaz was involved in the technology development and thus offered a minor share in the new startup firm fNIR Devices, LLC.

## Conflict of Interest Statement

fNIR Devices, LLC manufactures the optical brain imaging instrument and licensed IP and know-how from Drexel University. H. Ayaz was involved in the technology development and thus offered a minor share in the new startup firm fNIR Devices, LLC. The authors declare that the research was conducted in the absence of any commercial or financial relationships that could be construed as a potential conflict of interest.
